# 2-(2,4-Dichloro­phen­yl)-*N*-(1,5-dimethyl-3-oxo-2-phenyl-2,3-dihydro-1*H*-pyrazol-4-yl)acetamide

**DOI:** 10.1107/S1600536812049628

**Published:** 2012-12-08

**Authors:** Ray J. Butcher, Aneeka Mahan, P. S. Nayak, B. Narayana, H. S. Yathirajan

**Affiliations:** a Department of Chemistry, Howard University, 525 College Street NW, Washington, DC 20059, USA; b Lake Braddock Secondary School, 9200 Burke Lake Road, Burke, VA 22015, USA; c Department of Studies in Chemistry, Mangalore University, Mangalagangotri 574 199, India; d Department of Studies in Chemistry, University of Mysore, Manasagangotri, Mysore 570 006, India

## Abstract

In the crystal structure of the title compound, C_19_H_17_Cl_2_N_3_O_2_, the mol­ecules form dimers of the *R*_2_^2^(10) type through N—H⋯O hydrogen bonding. As a result of steric repulsion, the amide group is rotated with respect to both the dichloro­phenyl and 2,3-dihydro-1*H*-pyrazol-4-yl rings, making dihedral angles of 80.70 (13) and 64.82 (12)°, respectively. The dihedral angle between the dichloro­phenyl and 2,3-dihydro-1*H*-pyrazol-4-yl rings is 48.45 (5)° while that between the 2,3-dihydro-1*H*-pyrazol-4-yl and phenyl rings is 56.33 (6)°.

## Related literature

For a description of the Cambridge Structural Database, see: Allen (2002 ▶). For hydrogen-bond motifs, see: Bernstein *et al.* (1995 ▶). For *N*-substituted 2-aryl­acetamides and amides, see: Mijin & Marinkovic (2006 ▶); Mijin *et al.* (2008 ▶); Fun *et al.* (2011*a* ▶,*b* ▶); Fun, Shahani *et al.* (2012 ▶); Fun, Quah *et al.* (2012 ▶); Wu *et al.* (2008 ▶, 2010 ▶).
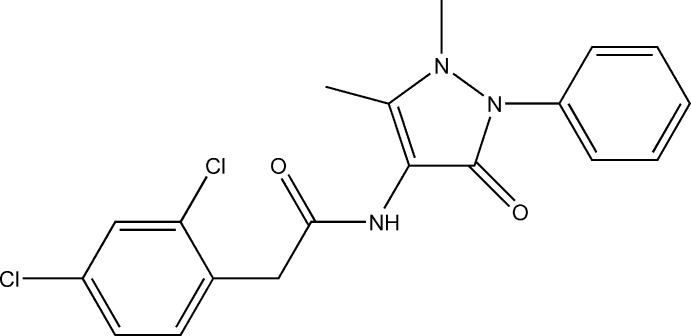


## Experimental

### 

#### Crystal data

C_19_H_17_Cl_2_N_3_O_2_
*M* *_r_* = 390.26
Monoclinic, 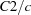

*a* = 25.1853 (5) Å
*b* = 8.18108 (9) Å
*c* = 21.0978 (4) Å
β = 119.772 (3)°
*V* = 3773.26 (16) Å^3^
*Z* = 8
Cu *K*α radiation
μ = 3.25 mm^−1^
*T* = 123 K
0.59 × 0.22 × 0.08 mm

#### Data collection

Agilent Xcalibur (Ruby, Gemini) diffractometer
Absorption correction: analytical [*CrysAlis PRO* (Agilent, 2011 ▶), based on expressions derived by Clark & Reid (1995 ▶)] *T*_min_ = 0.429, *T*_max_ = 0.804
12628 measured reflections
3849 independent reflections
3663 reflections with *I* > 2σ(*I*)
*R*_int_ = 0.027

#### Refinement

*R*[*F* ^2^ > 2σ(*F* ^2^)] = 0.035
*wR*(*F* ^2^) = 0.095
*S* = 1.05
3849 reflections
237 parameters
H-atom parameters constrained
Δρ_max_ = 0.57 e Å^−3^
Δρ_min_ = −0.30 e Å^−3^

### 

Data collection: *CrysAlis PRO* (Agilent, 2011 ▶); cell refinement: *CrysAlis PRO*; data reduction: *CrysAlis PRO*; program(s) used to solve structure: *SHELXS97* (Sheldrick, 2008 ▶); program(s) used to refine structure: *SHELXL97* (Sheldrick, 2008 ▶); molecular graphics: *SHELXTL* (Sheldrick, 2008 ▶); software used to prepare material for publication: *SHELXTL*.

## Supplementary Material

Crystal structure: contains datablock(s) I, global. DOI: 10.1107/S1600536812049628/bt6879sup1.cif

Structure factors: contains datablock(s) I. DOI: 10.1107/S1600536812049628/bt6879Isup2.hkl

Supplementary material file. DOI: 10.1107/S1600536812049628/bt6879Isup3.cml

Additional supplementary materials:  crystallographic information; 3D view; checkCIF report

## Figures and Tables

**Table 1:** Hydrogen-bond geometry (Å, °)

*D*—H⋯*A*	*D*—H	H⋯*A*	*D*⋯*A*	*D*—H⋯*A*
N1—H1*A*⋯O2^i^	0.88	1.92	2.7938 (15)	171

Symmetry code: (i) 
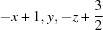
.

**Fig. 1.**
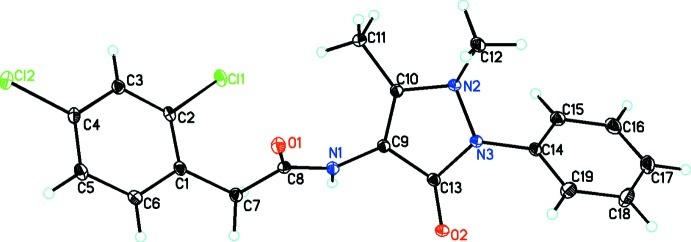
View of the molecular structure of the title compound with the atom-numbering scheme. Displacement ellipsoids are drawn at the 30% probability level for non-hydrogen atoms.

**Fig. 2.**
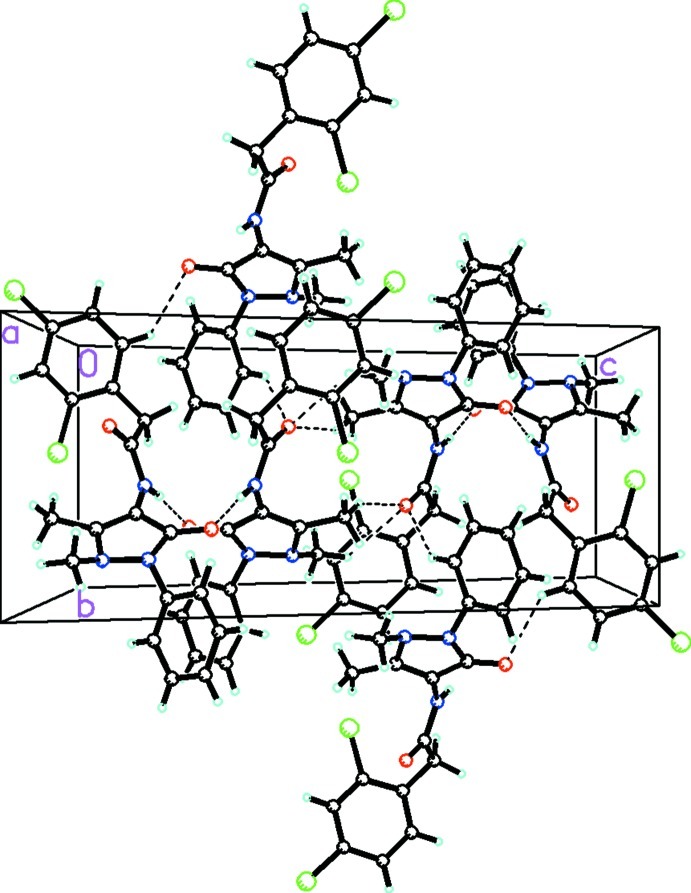
The packing view viewed along the a axis. Dashed lines indicate intermolecular N—H···O hydrogen bonds (see Table 1 for details).

## supplementary crystallographic information

### Comment

N-Substituted 2-arylacetamides are very interesting compounds because of their structural similarity to the lateral chain of natural benzylpenicillin (Mijin *et al.*, 2006, 2008). Amides are also used as ligands due to their excellent coordination abilities (Wu *et al.*, 2008, 2010). Crystal structures of some acetamide derivatives *viz*., (2E)-1-(2,5-dimethoxyphenyl)-3-(3-nitrophenyl)prop-2-en-1-one, *N*-(4-bromophenyl)-2-(naphthalen-1-yl)acetamide, *N*-(1,5-dimethyl-3-oxo-2-phenyl-2,3-dihydro-1*H*-pyrazol-4-yl)-2-\[4-(methylsulfanyl)phenyl]acetamide, *N*-(4-bromophenyl)-2-(4-chlorophenyl)acetamide (Fun *et al.*, 2011*a*; Fun *et al.*, 2011*b*; Fun, Shahani *et al.*, 2012; Fun, Quah *et al.*, 2012) have been reported. In view of the importance of amides we report herein the crystal structure of the title compound (I).

In the title compound, I, C_19_H_17_Cl_2_N_3_O_2_ the amide group is planar and through N—H···O hydrogen bonding to an adjoining molecule forms dimers of the *R*_2_^2^(10) type (Bernstein *et al.*, 1995). Due to steric repulsion the amide group is rotated with respect to both the dichlorophenyl and 2,3-dihydro-1*H*-pyrazol-4-yl rings with dihedral angles of 80.70 (13)° and 64.82 (12)° respectively. The dihedral angles between the three rings are 48.45 (5)° for the dichlorophenyl and 2,3-dihydro-1*H*-pyrazol-4-yl rings and and 56.33 (6)° for the 2,3-dihydro-1*H*-pyrazol-4-yl and phenyl rings, respectively. All other metrical parameters are in the normal ranges (Allen, 2002).

### Experimental

2,4-Dichlorophenylacetic acid (0.240 g, 1 mmol) and 4-aminoantipyrine (0.203 g, 1 mmol), 1-ethyl-3-(3-dimethylaminopropyl)-carbodiimide hydrochloride (1.0 g, 0.01 mol) and were dissolved in dichloromethane (20 ml). The mixture was stirred in presence of triethylamine at 273 K for about 3 h. The contents were poured into 100 ml of ice-cold aqueous hydrochloric acid with stirring, which was extracted thrice with dichloromethane. Organic layer was washed with saturated NaHCO_3_ solution and brine solution, dried and concentrated under reduced pressure to give the title compound (I). Single crystals were grown from methylene chloride by the slow evaporation method (m.p.: 473–475 K).

### Refinement

The H atoms were placed in calculated positions and refined in the riding mode: N—H = 0.88 Å, C—H = 0.95–0.99 Å with *U*_iso_(H) = 1.5*U*_eq_(C) for methyl H atoms and = 1.2*U*_eq_(O,C) for other H atoms.

### Crystal data

**Table:**

C_19_H_17_Cl_2_N_3_O_2_	*F*(000) = 1616
*M**_r_* = 390.26	*D*_x_ = 1.374 Mg m^−^^3^
Monoclinic, *C*2/*c*	Cu *K*α radiation, λ = 1.54184 Å
*a* = 25.1853 (5) Å	Cell parameters from 9236 reflections
*b* = 8.18108 (9) Å	θ = 3.5–75.5°
*c* = 21.0978 (4) Å	µ = 3.25 mm^−^^1^
β = 119.772 (3)°	*T* = 123 K
*V* = 3773.26 (16) Å^3^	Plate, colorless
*Z* = 8	0.59 × 0.22 × 0.08 mm

### Data collection

**Table:**

Agilent Xcalibur (Ruby, Gemini) diffractometer	3849 independent reflections
Radiation source: Enhance (Cu) X-ray Source	3663 reflections with *I* > 2σ(*I*)
Graphite monochromator	*R*_int_ = 0.027
Detector resolution: 10.5081 pixels mm^-1^	θ_max_ = 75.7°, θ_min_ = 4.0°
ω scans	*h* = −31→31
Absorption correction: analytical [*CrysAlis PRO* (Agilent, 2011), based on expressions derived by Clark & Reid (1995)]	*k* = −5→10
*T*_min_ = 0.429, *T*_max_ = 0.804	*l* = −24→26
12628 measured reflections	

### Refinement

**Table:**

Refinement on *F*^2^	Primary atom site location: structure-invariant direct methods
Least-squares matrix: full	Secondary atom site location: difference Fourier map
*R*[*F*^2^ > 2σ(*F*^2^)] = 0.035	Hydrogen site location: inferred from neighbouring sites
*wR*(*F*^2^) = 0.095	H-atom parameters constrained
*S* = 1.05	*w* = 1/[σ^2^(*F*_o_^2^) + (0.053*P*)^2^ + 3.0975*P*] where *P* = (*F*_o_^2^ + 2*F*_c_^2^)/3
3849 reflections	(Δ/σ)_max_ = 0.001
237 parameters	Δρ_max_ = 0.57 e Å^−^^3^
0 restraints	Δρ_min_ = −0.30 e Å^−^^3^

### Special details

**Table:**

Experimental. CrysAlisPro (Agilent Technologies, 2011) Analytical numeric absorption correction using a multifaceted crystal model based on expressions derived by Clark & Reid (1995).
Geometry. All e.s.d.'s (except the e.s.d. in the dihedral angle between two l.s. planes) are estimated using the full covariance matrix. The cell e.s.d.'s are taken into account individually in the estimation of e.s.d.'s in distances, angles and torsion angles; correlations between e.s.d.'s in cell parameters are only used when they are defined by crystal symmetry. An approximate (isotropic) treatment of cell e.s.d.'s is used for estimating e.s.d.'s involving l.s. planes.
Refinement. Refinement of *F*^2^ against ALL reflections. The weighted *R*-factor *wR* and goodness of fit *S* are based on *F*^2^, conventional *R*-factors *R* are based on *F*, with *F* set to zero for negative *F*^2^. The threshold expression of *F*^2^ > σ(*F*^2^) is used only for calculating *R*-factors(gt) *etc*. and is not relevant to the choice of reflections for refinement. *R*-factors based on *F*^2^ are statistically about twice as large as those based on *F*, and *R*- factors based on ALL data will be even larger.

### Fractional atomic coordinates and isotropic or equivalent isotropic displacement parameters (Å^2^)

**Table:**

	*x*	*y*	*z*	*U*_iso_*/*U*_eq_	
Cl1	0.322039 (15)	0.55288 (4)	0.50139 (2)	0.03074 (11)	
Cl2	0.26743 (2)	1.17963 (5)	0.42432 (2)	0.04088 (13)	
O1	0.48051 (5)	0.65179 (13)	0.60154 (6)	0.0275 (2)	
O2	0.58614 (5)	0.26875 (13)	0.77321 (5)	0.0281 (2)	
N1	0.46909 (5)	0.42833 (14)	0.65739 (6)	0.0225 (2)	
H1A	0.4505	0.3881	0.6799	0.027*	
N2	0.54781 (5)	0.15657 (15)	0.59681 (6)	0.0229 (2)	
N3	0.58823 (5)	0.16033 (15)	0.67299 (6)	0.0230 (2)	
C1	0.37725 (6)	0.80141 (17)	0.59726 (8)	0.0237 (3)	
C2	0.33397 (6)	0.75827 (17)	0.52636 (8)	0.0230 (3)	
C3	0.29970 (6)	0.87188 (18)	0.47298 (8)	0.0255 (3)	
H3A	0.2701	0.8385	0.4251	0.031*	
C4	0.30983 (7)	1.03615 (18)	0.49149 (8)	0.0263 (3)	
C5	0.35183 (7)	1.08602 (19)	0.56138 (9)	0.0299 (3)	
H5A	0.3579	1.1989	0.5735	0.036*	
C6	0.38487 (7)	0.96809 (19)	0.61340 (8)	0.0288 (3)	
H6A	0.4136	1.0018	0.6616	0.035*	
C7	0.41457 (7)	0.67467 (18)	0.65363 (8)	0.0276 (3)	
H7A	0.3868	0.5946	0.6574	0.033*	
H7B	0.4387	0.7282	0.7018	0.033*	
C8	0.45797 (6)	0.58490 (17)	0.63435 (7)	0.0217 (3)	
C9	0.50922 (6)	0.32646 (16)	0.64690 (7)	0.0212 (3)	
C10	0.50219 (6)	0.26811 (17)	0.58283 (7)	0.0220 (3)	
C11	0.45465 (7)	0.30761 (19)	0.50647 (8)	0.0278 (3)	
H11A	0.4249	0.3838	0.5071	0.042*	
H11B	0.4337	0.2070	0.4811	0.042*	
H11C	0.4741	0.3580	0.4809	0.042*	
C12	0.57913 (8)	0.1578 (2)	0.55384 (8)	0.0307 (3)	
H12A	0.5490	0.1455	0.5019	0.046*	
H12B	0.6084	0.0671	0.5694	0.046*	
H12C	0.6010	0.2615	0.5615	0.046*	
C13	0.56376 (6)	0.25707 (17)	0.70619 (7)	0.0219 (3)	
C14	0.62640 (6)	0.02126 (18)	0.70612 (7)	0.0236 (3)	
C15	0.60320 (8)	−0.13441 (19)	0.68234 (8)	0.0305 (3)	
H15A	0.5620	−0.1488	0.6446	0.037*	
C16	0.64078 (10)	−0.2690 (2)	0.71423 (10)	0.0428 (4)	
H16A	0.6256	−0.3760	0.6978	0.051*	
C17	0.70029 (10)	−0.2473 (3)	0.76984 (10)	0.0494 (5)	
H17A	0.7259	−0.3395	0.7917	0.059*	
C18	0.72255 (9)	−0.0917 (3)	0.79377 (10)	0.0491 (5)	
H18A	0.7634	−0.0777	0.8323	0.059*	
C19	0.68580 (7)	0.0448 (2)	0.76203 (9)	0.0352 (4)	
H19A	0.7012	0.1518	0.7784	0.042*	

### Atomic displacement parameters (Å^2^)

**Table:**

	*U*^11^	*U*^22^	*U*^33^	*U*^12^	*U*^13^	*U*^23^
Cl1	0.02802 (19)	0.01940 (18)	0.0432 (2)	−0.00059 (12)	0.01644 (16)	−0.00417 (13)
Cl2	0.0505 (2)	0.0273 (2)	0.0381 (2)	0.01167 (16)	0.01682 (19)	0.00933 (15)
O1	0.0318 (5)	0.0260 (5)	0.0317 (5)	0.0038 (4)	0.0210 (4)	0.0068 (4)
O2	0.0340 (5)	0.0323 (6)	0.0187 (5)	0.0082 (4)	0.0136 (4)	0.0003 (4)
N1	0.0281 (6)	0.0239 (6)	0.0229 (5)	0.0047 (5)	0.0182 (5)	0.0043 (4)
N2	0.0277 (6)	0.0261 (6)	0.0175 (5)	0.0045 (5)	0.0131 (5)	0.0010 (4)
N3	0.0270 (6)	0.0255 (6)	0.0182 (5)	0.0053 (5)	0.0126 (5)	0.0010 (4)
C1	0.0260 (6)	0.0233 (7)	0.0274 (7)	0.0042 (5)	0.0175 (6)	0.0007 (5)
C2	0.0237 (6)	0.0177 (6)	0.0317 (7)	0.0002 (5)	0.0169 (6)	−0.0022 (5)
C3	0.0228 (6)	0.0252 (7)	0.0283 (7)	0.0023 (5)	0.0125 (6)	−0.0013 (6)
C4	0.0279 (7)	0.0219 (7)	0.0313 (7)	0.0060 (5)	0.0163 (6)	0.0047 (5)
C5	0.0329 (7)	0.0199 (7)	0.0375 (8)	0.0013 (6)	0.0178 (6)	−0.0024 (6)
C6	0.0306 (7)	0.0265 (7)	0.0279 (7)	0.0021 (6)	0.0134 (6)	−0.0042 (6)
C7	0.0343 (7)	0.0284 (7)	0.0265 (7)	0.0083 (6)	0.0201 (6)	0.0035 (6)
C8	0.0239 (6)	0.0239 (7)	0.0182 (6)	0.0027 (5)	0.0112 (5)	0.0012 (5)
C9	0.0257 (6)	0.0212 (6)	0.0210 (6)	0.0022 (5)	0.0149 (5)	0.0028 (5)
C10	0.0259 (6)	0.0212 (6)	0.0222 (6)	0.0017 (5)	0.0143 (5)	0.0023 (5)
C11	0.0311 (7)	0.0315 (8)	0.0200 (6)	0.0054 (6)	0.0122 (6)	0.0016 (5)
C12	0.0407 (8)	0.0339 (8)	0.0276 (7)	0.0103 (6)	0.0247 (7)	0.0049 (6)
C13	0.0265 (6)	0.0215 (6)	0.0219 (6)	0.0011 (5)	0.0152 (5)	0.0005 (5)
C14	0.0269 (7)	0.0276 (7)	0.0212 (6)	0.0067 (5)	0.0157 (6)	0.0029 (5)
C15	0.0384 (8)	0.0285 (8)	0.0274 (7)	0.0029 (6)	0.0184 (6)	0.0018 (6)
C16	0.0682 (12)	0.0297 (8)	0.0372 (9)	0.0139 (8)	0.0312 (9)	0.0059 (7)
C17	0.0635 (12)	0.0501 (11)	0.0374 (9)	0.0336 (10)	0.0273 (9)	0.0158 (8)
C18	0.0354 (9)	0.0679 (13)	0.0355 (9)	0.0211 (9)	0.0112 (7)	0.0108 (9)
C19	0.0299 (7)	0.0423 (9)	0.0295 (7)	0.0038 (7)	0.0119 (6)	0.0006 (7)

### Geometric parameters (Å, º)

**Table:**

Cl1—C2	1.7419 (14)	C7—H7A	0.9900
Cl2—C4	1.7388 (15)	C7—H7B	0.9900
O1—C8	1.2210 (17)	C9—C10	1.3597 (19)
O2—C13	1.2390 (17)	C9—C13	1.4378 (19)
N1—C8	1.3492 (18)	C10—C11	1.4892 (19)
N1—C9	1.4097 (17)	C11—H11A	0.9800
N1—H1A	0.8800	C11—H11B	0.9800
N2—C10	1.3796 (18)	C11—H11C	0.9800
N2—N3	1.4128 (15)	C12—H12A	0.9800
N2—C12	1.4678 (17)	C12—H12B	0.9800
N3—C13	1.3874 (17)	C12—H12C	0.9800
N3—C14	1.4282 (18)	C14—C19	1.383 (2)
C1—C2	1.390 (2)	C14—C15	1.387 (2)
C1—C6	1.395 (2)	C15—C16	1.388 (2)
C1—C7	1.504 (2)	C15—H15A	0.9500
C2—C3	1.383 (2)	C16—C17	1.381 (3)
C3—C4	1.387 (2)	C16—H16A	0.9500
C3—H3A	0.9500	C17—C18	1.382 (3)
C4—C5	1.382 (2)	C17—H17A	0.9500
C5—C6	1.386 (2)	C18—C19	1.392 (3)
C5—H5A	0.9500	C18—H18A	0.9500
C6—H6A	0.9500	C19—H19A	0.9500
C7—C8	1.5302 (18)		
			
C8—N1—C9	122.77 (11)	N1—C9—C13	123.13 (12)
C8—N1—H1A	118.6	C9—C10—N2	109.64 (12)
C9—N1—H1A	118.6	C9—C10—C11	129.58 (13)
C10—N2—N3	106.37 (10)	N2—C10—C11	120.77 (12)
C10—N2—C12	120.64 (11)	C10—C11—H11A	109.5
N3—N2—C12	113.45 (11)	C10—C11—H11B	109.5
C13—N3—N2	109.70 (11)	H11A—C11—H11B	109.5
C13—N3—C14	124.55 (11)	C10—C11—H11C	109.5
N2—N3—C14	117.97 (11)	H11A—C11—H11C	109.5
C2—C1—C6	116.75 (13)	H11B—C11—H11C	109.5
C2—C1—C7	121.62 (13)	N2—C12—H12A	109.5
C6—C1—C7	121.63 (13)	N2—C12—H12B	109.5
C3—C2—C1	123.01 (13)	H12A—C12—H12B	109.5
C3—C2—Cl1	117.25 (11)	N2—C12—H12C	109.5
C1—C2—Cl1	119.73 (11)	H12A—C12—H12C	109.5
C2—C3—C4	117.95 (13)	H12B—C12—H12C	109.5
C2—C3—H3A	121.0	O2—C13—N3	123.76 (13)
C4—C3—H3A	121.0	O2—C13—C9	131.24 (13)
C5—C4—C3	121.51 (14)	N3—C13—C9	104.95 (11)
C5—C4—Cl2	120.34 (12)	C19—C14—C15	121.28 (14)
C3—C4—Cl2	118.15 (12)	C19—C14—N3	119.11 (14)
C4—C5—C6	118.67 (14)	C15—C14—N3	119.61 (13)
C4—C5—H5A	120.7	C14—C15—C16	119.30 (16)
C6—C5—H5A	120.7	C14—C15—H15A	120.4
C5—C6—C1	122.10 (14)	C16—C15—H15A	120.4
C5—C6—H6A	119.0	C17—C16—C15	120.07 (18)
C1—C6—H6A	119.0	C17—C16—H16A	120.0
C1—C7—C8	111.65 (11)	C15—C16—H16A	120.0
C1—C7—H7A	109.3	C16—C17—C18	120.05 (17)
C8—C7—H7A	109.3	C16—C17—H17A	120.0
C1—C7—H7B	109.3	C18—C17—H17A	120.0
C8—C7—H7B	109.3	C17—C18—C19	120.76 (18)
H7A—C7—H7B	108.0	C17—C18—H18A	119.6
O1—C8—N1	123.92 (12)	C19—C18—H18A	119.6
O1—C8—C7	122.02 (13)	C14—C19—C18	118.54 (17)
N1—C8—C7	114.06 (12)	C14—C19—H19A	120.7
C10—C9—N1	127.88 (13)	C18—C19—H19A	120.7
C10—C9—C13	108.83 (12)		
			
C10—N2—N3—C13	7.36 (15)	N1—C9—C10—C11	7.1 (2)
C12—N2—N3—C13	142.30 (12)	C13—C9—C10—C11	−177.35 (14)
C10—N2—N3—C14	158.08 (12)	N3—N2—C10—C9	−6.32 (15)
C12—N2—N3—C14	−66.98 (16)	C12—N2—C10—C9	−137.32 (14)
C6—C1—C2—C3	0.5 (2)	N3—N2—C10—C11	174.04 (12)
C7—C1—C2—C3	−178.87 (13)	C12—N2—C10—C11	43.0 (2)
C6—C1—C2—Cl1	179.45 (10)	N2—N3—C13—O2	172.17 (13)
C7—C1—C2—Cl1	0.12 (18)	C14—N3—C13—O2	23.8 (2)
C1—C2—C3—C4	0.7 (2)	N2—N3—C13—C9	−5.45 (15)
Cl1—C2—C3—C4	−178.31 (10)	C14—N3—C13—C9	−153.82 (13)
C2—C3—C4—C5	−1.4 (2)	C10—C9—C13—O2	−175.84 (15)
C2—C3—C4—Cl2	179.34 (10)	N1—C9—C13—O2	0.0 (2)
C3—C4—C5—C6	0.9 (2)	C10—C9—C13—N3	1.53 (15)
Cl2—C4—C5—C6	−179.86 (11)	N1—C9—C13—N3	177.34 (12)
C4—C5—C6—C1	0.4 (2)	C13—N3—C14—C19	−72.00 (19)
C2—C1—C6—C5	−1.0 (2)	N2—N3—C14—C19	142.00 (13)
C7—C1—C6—C5	178.33 (14)	C13—N3—C14—C15	107.33 (16)
C2—C1—C7—C8	66.19 (18)	N2—N3—C14—C15	−38.67 (17)
C6—C1—C7—C8	−113.11 (15)	C19—C14—C15—C16	−1.4 (2)
C9—N1—C8—O1	1.2 (2)	N3—C14—C15—C16	179.28 (14)
C9—N1—C8—C7	−178.10 (13)	C14—C15—C16—C17	1.1 (2)
C1—C7—C8—O1	31.9 (2)	C15—C16—C17—C18	−0.1 (3)
C1—C7—C8—N1	−148.72 (13)	C16—C17—C18—C19	−0.5 (3)
C8—N1—C9—C10	−67.1 (2)	C15—C14—C19—C18	0.8 (2)
C8—N1—C9—C13	117.97 (15)	N3—C14—C19—C18	−179.91 (15)
N1—C9—C10—N2	−172.50 (13)	C17—C18—C19—C14	0.2 (3)
C13—C9—C10—N2	3.05 (16)		

### Hydrogen-bond geometry (Å, º)

**Table:**

*D*—H···*A*	*D*—H	H···*A*	*D*···*A*	*D*—H···*A*
N1—H1*A*···O2^i^	0.88	1.92	2.7938 (15)	171

Symmetry code: (i) −*x*+1, *y*, −*z*+3/2.
